# Factors associated with blood culture positivity in patients with complicated skin and skin structure infection—a population-based study

**DOI:** 10.1007/s10096-019-03560-9

**Published:** 2019-04-22

**Authors:** Mika Halavaara, Iiro H. Jääskeläinen, Lars Hagberg, Asko Järvinen

**Affiliations:** 1 Department of Infectious Diseases, Inflammation Center, Helsinki University Hospital and University of Helsinki, P.O. Box 372, 00029 HUS Helsinki, Finland; 2 Department of Infectious Diseases, Institute of Biomedicine, Sahlgrenska Academy, University of Gothenburg, Gothenburg, Sweden

**Keywords:** Skin and soft tissue infection, Cellulitis, C-reactive protein, Bacteremia, Abscess, Bloodstream infection

## Abstract

Skin and skin structure infection (SSSI) is classified as complicated (cSSSI) if it involves deep subcutaneous tissue or requires surgery. Factors associated with blood culture sampling and bacteremia have not been established in patients with cSSSI. Moreover, the benefit of information acquired from positive blood culture is unknown. The aim of this study was to address these important issues. In this retrospective population-based study from two Nordic cities, a total of 460 patients with cSSSI were included. Blood cultures were drawn from 258 (56.1%) patients and they were positive in 61 (23.6%) of them. Factors found to be associated with more blood culture sampling in multivariate analysis were diabetes, duration of symptoms shorter than 2 days and higher C-reactive protein (CRP) level. Whereas factors associated with less frequent blood culture sampling were peripheral vascular disease and a surgical wound infection. In patients from whom blood cultures were taken, alcohol abuse was the only factor associated with culture positivity, as CRP level was not. Patients with a positive blood culture had antibiotic streamlining more often than non-bacteremic patients. A high rate of blood culture positivity in patients with cSSSI was observed. Factors related to more frequent blood culture sampling were different from those associated with a positive culture.

## Introduction

Skin and skin structure infections (SSSIs) are among the most common bacterial infections in patients presenting in emergency rooms and their incidence is rising [1–3]. In 1998, FDA classified SSSI as complicated (cSSSI) if it involves deep subcutaneous tissues or requires surgery [4]. Although initially designed for the clinical trials, the umbrella term cSSSI is still useful in the detection of the most severe forms of SSSIs [5].

Blood cultures are not routinely recommended for patients with SSSI [6, 7]. This is mainly because positive findings have been rare [8] and have only seldom affected antibiotic treatment [9]. However, this might not be generalizable to all SSSIs since the rate of bacteremia has been reported to increase in more severe cases. Whereas blood culture positivity of 4.6% has been reported in erysipelas, it was 7.9% in cellulitis [10] and even higher rate of 11.9% was reported in a recent European survey on cSSSI [11]. Factors associated with bacteremia in cSSSI have not been studied, but in less severe SSSI comorbidity [12] in another study, up to 11 patient factors, including male gender and older age, were linked to bacteremia [13].

We conducted a population-based study including 460 patients with cSSSI from two Nordic cities and reported that at least 13.3% of patients had a bloodstream infection with equal yield of one fourth of samples being positive in both study sites [14]. Male gender and cellulitis were associated with blood culture sampling and bacteremia with later clinical stability [14, 15]. In the present study, we analysed further from the same real-life setup factors predicting and associated with blood culture positivity and how the knowledge of blood culture positivity affected the treatment.

## Materials and methods

The study design was a retrospective observational cohort study. All adult patients hospitalised for cSSSI within a 4-year period 2008–2011 in Helsinki University Hospital and Helsinki City Hospital in Helsinki, Finland (604,000 inhabitants), and Sahlgrenska University Hospital in Gothenburg, Sweden (525,000 inhabitants), were included in the study [14]. Patients were recognised using *International Classification of Diseases* (ICD-10) codes and demographic and clinical data were collected from the medical records. These hospitals have the only emergency departments in their catchment areas why virtually all hospitalised patients with cSSSI have been included enabling the population-based approach. The prevalence of methicillin-resistant *Staphylococcus aureus* was 2.8% in Finland [16] and 0.8% in Sweden in 2011 [17].

Detailed study protocol is presented in the primary publication of this study [14]. In short, to be included, patients were required to have infection affecting deeper soft tissue (e.g. cellulitis or fasciitis), infection requiring significant surgical intervention, infection which developed on a lower extremity in a patient with diabetes mellitus or peripheral vascular disease or to have a major abscess or an infected ulcer. Patients also had to have at least one systemic sign of infection: temperature > 38 °C or < 36 °C or white blood cell count > 10,000/mm^3^ or < 4000/mm^3^.

### Study definitions and statistical analyses

Microbial diagnosis was obtained by blood culture, bacterial culture of tissue or superficial swabs in routine cultures. Cellulitis/fasciitis was defined as an infection without abscess, diabetic foot/leg ulcer or peripheral vascular disease ulcer. The evaluation of clinical stability was based on improvement of vital signs and decrease of fever. Streamlining was defined as change of antibiotic therapy to pathogen specific one. Microbes from normal cutaneous flora (e.g. coagulase-negative staphylococci) were generally not considered as pathogens in blood cultures and an infectious disease specialist assessed each case. Antibiotic treatment prescribed before admission or before the fulfilment of cSSSI criteria during the hospitalisation was recorded.

Categorical variables were summarised using counts and percentages. Continuous variables were summarised using means and standard deviation (SD) or median, interquartile range (IQR), or range if subgroup was small. In univariate analysis, the difference between two groups was compared using chi-square test or Fisher’s exact test, as appropriate. Continuous variables were analysed using a two-sample *t* test or Mann-Whitney *U* test if variables were not normally distributed. Odds ratio (OR) was calculated with 95% confidence interval (CI). Multivariate logistic regression was performed including variables that were clinically relevant, had univariate *p* values less than 0.15 and were not multicollinear. *P* value < 0.05 was considered significant. SPSS version 22.0 (SPSS Inc., Chicago, IL, USA) was used for statistical analyses.

Factors associated with blood culture sampling were analysed also using multivariate logistic regression analysis and factors associated with bacteremia were analysed by comparing blood culture positive patients with blood culture negative patients. This approach differs from the analysis performed in the previous publications of this material [14, 15].

## Results

### Blood culture findings

In total, 460 patients with cSSSI were included. Blood cultures were drawn from 258 (51.6%) patients and they were positive in 61 (23.6%). Although there was some heterogeneity in the patient populations between the two centres [18], proportion of positive blood cultures was almost equal, in Helsinki 22.9% and 25% in Gothenburg. Blood culture isolates were *Streptococcus pyogenes* 19 cases (31.1%), *Staphylococcus aureus* 19 (31.1%), non-A beta-hemolytic streptococci 12 (19.7%), *Streptococcus pneumoniae* 1 (1.6%), *Enterobacteriaceae* spp. 1 (1.6%), polymicrobial 5 (8.2%) and unknown (4/6.6%).

### Factors associated with blood culture sampling

Patients from whom blood cultures were drawn (*n* = 258) were compared to those from whom blood cultures were not drawn (*n* = 202) for this analysis (Table 1).

**Table 1:** Demographics, clinical features and laboratory findings of 460 patients with cSSSI according to if blood culture sample was taken or not taken. Data is shown as number of patients (%) in each column

Variable	All (*n* = 460)	Blood cultures drawn (*n* = 258)	Blood cultures not drawn (*n* = 202)	OR (95% CI)	*P* value	Multivariate logistic regression analysis	
						OR (95% CI)	*P* value
Male gender	280 (60.9)	168 (65.1)	112 (55.4)	1.5 (1.0–2.2)	0.035	1.2 (0.8–1.9)	0.337
Age, years [mean (SD)]	67.4 (18.1)	66.8 (17.5)	68.1 (19.0)		0.465		
Diabetes	187 (40.7)	120 (46.5)	67 (33.2)	1.7 (1.2–2.6)	0.004	1.9 (1.2–2.9)	0.008
Peripheral vascular disease	135 (29.3)	62 (24.0)	73 (36.1)	0.6 (0.4–0.8)	0.005	0.5 (0.3–0.8)	0.007
Congestive heart disease	43 (9.3)	23 (8.9)	20 (9.9)	0.9 (0.5–1.7)	0.718		
Respiratory disease	34 (7.4)	22 (8.5)	12 (5.9)	1.5 (0.7–3.1)	0.293		
Chronic renal disease	32 (7.0)	21 (8.1)	11 (5.4)	1.5 (0.7–3.3)	0.260		
Liver disease	23 (5.0)	14 (5.4)	9 (4.5)	1.2 (0.5–2.9)	0.673		
HIV infection	7 (1.5)	5 (1.9)	2 (1.0)	2.0 (0.4–10.3)	0.474		
Any disease with immune system impairment	14 (3.0)	6 (2.3)	8 (4.0)	0.6 (0.2–1.7)	0.311		
Malignancy	36 (7.8)	19 (7.4)	17 (8.4)	0.9 (0.4–1.7)	0.677		
Alcohol abuse	40 (8.7)	28 (10.9)	12 (5.9)	1.9 (1.0–3.9)	0.064	1.4 (0.6–3.1)	0.475
Intravenous drug use	32 (7.0)	15 (5.8)	17 (8.4)	0.7 (0.3–1.4)	0.276		
No. of co-morbidities ≥ 2	171 (37.2)	98 (38.0)	73 (36.1)	1.1 (0.7–1.6)	0.684		
Hospitalisation < 3 months	84 (18.3)	44 (17.1)	40 (19.8)	0.8 (0.5–1.3)	0.449		
Invasive surgery < 3 months	71 (15.4)	33 (12.8)	38 (18.8)	0.6 (0.4–1.1)	0.076		
Antibiotic treatment before dg	128 (27.8)	58 (22.5)	70 (34.7)	0.5 (0.4–0.8)	0.004	0.7 (0.4–1.1)	0.115
Abscess	183 (39.8)	91 (35.3)	92 (45.5)	0.7 (0.4–1.0)	0.025		
Cellulitis/fasciitis (no abscess or ulcer)	193 (42.0)	128 (49.6)	65 (32.2)	2.1 (1.4–3.0)	< 0.001	1.6 (1.0–2.5)	0.052
Decubitus or pressure ulcer	14 (3.0)	6 (2.3)	8 (4.0)	0.6 (0.2–1.7)	0.311		
Diabetic foot/leg ulcer	66 (14.3)	31 (12.0)	35 (17.3)	0.7 (0.4–1.1)	0.107		
Peripheral vascular disease ulcer	53 (11.5)	21 (8.1)	32 (15.8)	0.5 (0.3–0.8)	0.010		
Post-surgical wound	79 (17.2)	35 (13.6)	44 (21.8)	0.6 (0.3–0.9)	0.020	0.4 (0.2–0.8)	0.005
Post-traumatic wound	50 (10.9)	30 (11.6)	20 (9.9)	1.2 (0.7–2.2)	0.555		
Anatomical site of the infection							
Head	5 (1.1)	4 (1.6)	1 (0.5)	3.2 (0.4–28.5)	0.391		
Hand	9 (2.0)	4 (1.6)	5 (2.5)	0.6 (0.2–2.3)	0.515		
Trunk	142 (30.9)	73 (28.3)	69 (34.2)	0.8 (0.5–1.1)	0.177		
Upper extremities	52 (11.3)	33 (12.8)	19 (9.4)	1.4 (0.8–2.6)	0.255		
Lower extremities	284 (61.7)	167 (64.7)	117 (57.9)	1.3 (0.9–1.9)	0.136	1.4 (0.8–2.2)	0.214
Duration of symptoms before the diagnosis							
< 2 days	130 (28.3)	97 (37.6)	33 (16.3)	3.1 (2.0–4.8)	< 0.001	3.0 (1.8–5.2) ^1^	< 0.001
2–7 days	230 (50.0)	129 (50)	101 (50.0)	1.0 (0.7–1.4)	> 0.99		
> 7 days	90 (19.6)	29 (11.2)	61 (30.2)	0.3 (0.2–0.5)	< 0.001		
C-reactive protein (CRP) mg/L (*n* = 425)							
1st day CRP > 150	214 (50.4)	139 (57.0)	75 (41.4)	1.9 (1.3–2.8)	0.002	1.8 (1.2–2.8)	0.006
1st day CRP [median (IQR)]	157 (81–252)	181 (91–266)	130 (73–210)		0.003		

*OR* odds ratio, *CI* confidence interval, *HIV* human immunodeficiency virus, *No.* number, *Dg* diagnosis, *IQR* interquartile range

^1^In multivariate logistic regression analysis, this variable is included as dichotomic (i.e. symptoms shorter than 2 days compared to longer than 2 days)

In logistic regression analysis, diabetes (OR 1.9, *P* = 0.008), peripheral vascular disease (OR 0.5, *P* = 0.007), post-surgical wound infection (OR 0.4, *P* = 0.005), symptoms shorter than 2 days (OR 3.0, *P* < 0.001) and CRP over 150 mg/L on the first day (OR 1.8, *P* = 0.006) were significantly associated with blood culture sampling (Table 1).

### Factors associated with blood culture positivity

To analyse factors associated with blood culture positivity, we compared patients with a positive blood culture (*n* = 61) to patients with a negative blood culture (*n* = 197).

Results of comparisons between the groups are shown in Table 2. Neither CRP measured at the time of the diagnosis of cSSSI nor the highest CRP during the hospital stay was associated with blood culture positivity (Table 2). In multivariate logistic regression analysis, only factor associated significantly with bacteremia was alcohol abuse (OR 5.5, *P* < 0.001).

**Table 2:** Difference in demographics and clinical features of patients with blood culture positivity and negativity among 258 patients with cSSSI and blood culture sample taken. Data is shown as number of patients (%) in each column

Variable	All (*n* = 258)	Positive blood cultures (*n* = 61)	Negative blood cultures (*n* = 197)	OR (95% CI)	*P* value	Multivariate logistic regression analysis	
						OR (95% CI)	*P* value
Demographics and co-morbidities							
Male gender	168 (65.1)	42 (68.9)	126 (64.0)	1.2 (0.7–2.3)	0.484		
Age > 60 years	165 (64.0)	40 (65.6)	125 (63.5)	1.1 (0.6–2.0)	0.763		
Age, years [mean (SD)]	66.8 (17.5)	66.2 (16.4)	67.0 (17.9)		0.734		
Diabetes	120 (46.5)	27 (44.3)	93 (47.2)	0.9 (0.5–1.6)	0.687		
Peripheral vascular disease	62 (24.0)	15 (24.6)	47 (23.9)	1.0 (0.5–2.0)	0.907		
Congestive heart disease	23 (8.9)	2 (3.3)	21 (10.7)	0.3 (0.1–1.2)	0.077	0.2 (0.05–1.04)	0.057
Respiratory disease	22 (8.5)	9 (14.8)	13 (6.6)	2.5 (1.0–6.0)	0.046	2.2 (0.8–5.8)	0.133
Chronic renal disease	21 (8.1)	3 (4.9)	18 (9.1)	0.5 (0.1–1.8)	0.423		
Liver disease	14 (5.4)	6 (9.8)	8 (4.1)	2.6 (0.9–7.7)	0.104		
HIV infection	5 (1.9)	1 (1.6)	4 (2.0)	0.8 (0.1–7.3)	> 0.999		
Any disease with immune system impairment	6 (2.3)	3 (4.9)	3 (1.5)	3.3 (0.7–17.0)	0.146		
Cancer/malignancy	19 (7.4)	5 (8.2)	14 (7.1)	1.2 (0.4–3.4)	0.781		
Alcohol abuse	28 (10.9)	15 (24.6)	13 (6.6)	4.6 (2.1–10.4)	< 0.001	5.5 (2.3–13.2)	< 0.001
Intravenous drug use	15 (5.8)	3 (4.9)	12 (6.1)	0.8 (0.2–2.9)	> 0.999		
No. of co-morbidities ≥ 2	98 (38.0)	26 (42.6)	72 (36.5)	1.3 (0.7–2.3)	0.393		
Hospitalisation < 3 months	44 (17.1)	12 (19.7)	32 (16.2)	1.3 (0.6–2.6)	0.534		
Invasive surgery < 3 months	33 (12.8)	7 (11.5)	26 (13.2)	0.9 (0.4–2.1)	0.725		
Antibiotic treatment before dg	58 (22.5)	14 (23)	44 (22.3)	1.0 (0.5–2.0)	0.920		
Clinical features							
Abscess	91 (35.3)	15 (24.6)	76 (38.6)	0.5 (0.3–1.0)	0.046		
Cellulitis/fasciitis	128 (49.6)	37 (60.7)	91 (46.2)	1.8 (1.0–3.2)	0.048	1.6 (0.8–3.2)	0.162
Decubitus or pressure ulcer	6 (2.3)	1 (1.6)	5 (2.5)	0.6 (0.1–5.6)	> 0.999		
Diabetic foot/leg ulcer	31 (12.0)	9 (14.8)	22 (11.2)	1.4 (0.6–3.2)	0.452		
Peripheral vascular disease ulcer	21 (8.1)	3 (4.9)	18 (9.1)	0.5 (0.1–1.8)	0.423		
Post-surgical wound	35 (13.6)	5 (8.2)	30 (15.2)	0.5 (0.2–1.3)	0.161		
Post-traumatic wound	30 (11.6)	10 (16.4)	20 (10.2)	1.7 (0.8–3.9)	0.184		
Anatomical site of the infection							
Head	4 (1.6)	2 (3.3)	2 (1.0)	3.3 (0.5–24.0)	0.238		
Hand	4 (1.6)	1 (1.6)	3 (1.5)	1.000	> 0.999		
Trunk	73 (28.3)	10 (16.4)	63 (32.0)	0.4 (0.2–0.9)	0.018		
Upper extremities	33 (12.8)	12 (19.7)	21 (10.7)	2.1 (0.9–4.5)	0.066		
Lower extremities	167 (64.7)	45 (73.8)	122 (61.9)	1.7 (0.9–3.3)	0.091	2.0 (0.98–4.2)	0.055
Duration of symptoms before the diagnosis							
< 2 days	97 (37.6)	28 (45.9)	69 (35.0)	1.6 (0.9–2.8)	0.125	1.3 (0.7–2.6)^1^	0.425
2–7 days	129 (50)	21 (34.4)	108 (54.8)	0.4 (0.2–0.8)	0.005		
**>** 7 days	29 (11.2)	11 (18.0)	18 (9.1)	2.2 (1.0-4.9)	0.055		
C-reactive protein (CRP) mg/L							
1st day CRP count [median (IQR)] (*n* = 244)	181 (91–266)	201 (97–286)	170 (89–261)		0.410		
Highest CRP count [median (IQR)] (*n* = 256)	240 (156–320)	243 (166–331)	240 (150–311)		0.465		

*OR* odds ratio, *CI* confidence interval, *SD* standard deviation, *No.* number, *Dg* diagnosis, *CRP* C-reactive protein, *IQR* interquartile range

^1^In multivariate logistic regression analysis, this variable is included as dichotomic (i.e. symptoms shorter than 2 days compared to longer than 2 days)

### Clinical endpoints in blood culture positivity

Bacteremic patients (*n* = 61) were less likely to reach clinical stability within 3 days and they were more often admitted to intensive care unit and had significantly longer hospital stay than blood culture negative patients (Table 3). In addition, 23.3% of blood culture positive patients had antibiotic treatment streamlined as compared to 6.3% of culture negative patients (*P* = 0.0002).

**Table 3:** Outcome of 258 cSSSI patients from whom blood cultures were drawn. Data is shown as number of patients (%) in each column

Variable	All (*n* = 258)	Positive blood cultures (*n* = 61)	Negative blood cultures (*n* = 197)	Odds ratio (95% CI)	*P* value
Clinical stability within 3 days (*n* = 223)	105 (47.1)	15 (30)	90 (52)	0.4 (0.2–0.8)	0.006
Admission to ICU	51 (19.8)	20 (32.8)	31 (15.7)	2.6 (1.4–5.0)	0.003
Surgical intervention after the diagnosis of cSSSI	129 (50)	34 (55.7)	95 (48.2)	1.4 (0.8–2.4)	0.305
30-day mortality	16 (6.2)	4 (6.6)	12 (6.1)	1.1 (0.3–3.5)	> 0.999
Streamlining (*n* = 251)	26 (10.4)	14 (23.3)	12 (6.3)	4.5 (2.0–10.5)	< 0.001
Duration of antimicrobial treatment, days [median (IQR)] (*n* = 255)	21 (12–38)	26 (11.5–46.8)	20 (12–38)		0.191
Length of hospital stay, days [median (IQR)] (*n* = 228)	15 (8–29)	19.5 (13–45.3)	13 (7–23)		< 0.001

*CI* confidence interval, *IQR* interquartile range

## Discussion

In this population-based study, we observed that 23.6% of the cSSSI patients from whom blood culture was taken had bacteremia. Streptococci and *Staphylococcus aureus* corresponded for 84% of cases. Bacteremia was associated with later clinical stability, more ICU admissions and more common streamlining. Higher CRP was linked to more common blood culture sampling, but not to culture positivity. Diabetes and duration of symptoms shorter than 2 days were observed to increase the likelihood of blood culture sampling, but only alcoholism increased the likelihood of blood culture positivity.

Blood culture positivity reported here was higher than 11.6% in a multi-centre study from Central and Southern Europe, which had a sampling rate of 53% [11]. The most evident explanation is that our patient material was more severe. Blood culture positivity rate in less severe SSSIs like erysipelas or cellulitis has been reported to be 4.6–9% [10, 19].

In previous study on this patient material, it was observed that patients who had blood cultures drawn had higher mortality suggesting that clinicians ordered blood cultures from sicker patients [14]. Although the blood culture drawing rate differed in the two centres, the rate of positive findings was equal suggesting that blood cultures should most probably be taken with lower threshold in cSSSI.

Diabetes and higher CRP provoked clinicians to order blood culture sampling in accordance with data in erysipelas [19] and uncomplicated cellulitis [20]. In contrast to one previous study, prior antibiotic treatment was not negatively associated with positive blood culture findings but it resulted in less common (45.3%) sampling than compared to patients without prior antibiotics (54.7%, *P* = 0.004) [21]. Alcohol abuse had a striking association with blood culture positivity, as of patients with a history of alcohol abuse 53.6% had bacteremia as compared to 20% of those who did not. Similarly, in patients with uncomplicated cellulitis, alcohol abuse was the only discriminant patient characteristic associated with bacteremia [20].

Beta-hemolytic streptococci and *Staphylococcus aureus* were not only the most common blood culture findings but they also constituted 64% of all cSSSI cases in which the aetiology was verified. Whereas we observed only one case of gram-negative monobacteremia, Van Daalen et al. found more gram-negative bacteria than *Staphylococcus aureus* [12] and Peralta et al. observed a gram-negative aetiology in 24.6% of bacteremias [21]. Accordingly, in a systematic review of patients with cellulitis and erysipelas, gram-negative bacteria were concluded to be at least as common as *S. aureus* in blood cultures in cellulitis [10]. These differences might be explained by difference in patient selection in these studies.

Our results contradict the view that blood cultures would not be useful because in complicated cellulitis they rarely had an effect on antibiotic therapy [9]. In our material, antibiotic treatment was streamlined more often in bacteremic than in non-bacteremic patients (23.3% versus 6.3%, *P* < 0.001). Accordingly, a change in the antibiotic treatment was recorded in 49% of patients with lower limb cellulitis after blood culture results became positive [21]. Furthermore, in countries with higher antibiotic resistance, the importance of blood culture-directed therapy has been pointed out [22].

Blood culture positivity was linked to later clinical stability, which without culture result might lead to a premature change of antibiotic treatment to more broad-spectrum. However, positive blood culture was not linked to longer antibiotic treatment or higher mortality as has been reported previously [11]. The likely explanation to these differences is the low number of bacteremic patients and virtual lack of gram-negative and resistant bacteria in our study.

The strength of this study is its population-based nature, although some patients may have been unrecognised due to coding inaccuracy. Major limitations of this study are due to its retrospective nature. Data was collected from medical records, which left missing data in some parameters. Fifty-six percent of patients were subjected to blood culture sampling and were included in the analysis of factors associated with bacteremia, creating an inevitable selection bias.

In this population-based study in cSSSI, we observed that a positive blood culture was more common than previously reported and affected 23.6% of patients with blood culture sampled. Factors linked to higher blood culture sampling rate were not generally related to higher positive finding yield. A clear benefit of blood culture positivity on patient management was shown in more frequent antibiotic streamlining and knowledge of later clinical stability.

## References

[1] Raff AB, Kroshinsky D (2016). Cellulitis: a review. JAMA.

[2] Edelsberg J, Taneja C, Zervos M, Haque N, Moore C, Reyes K, Spalding J, Jiang J, Oster G (2009). Trends in US hospital admissions for skin and soft tissue infections. Emerg Infect Dis.

[3] Kaye KS, Patel DA, Stephens JM, Khachatryan A, Patel A, Johnson K (2015). Rising United States hospital admissions for acute bacterial skin and skin structure infections: recent trends and economic impact. PLoS One.

[4] Center for Drug Evaluation and Research, Food and Drug Administration (1998) Guidance for industry: uncomplicated and complicated skin and skin structure infections - developing antimicrobial drugs for treatment. Washington, DC: Food and Drug Administration, US Department of Health and Human Services

[5] Dryden MS (2010). Complicated skin and soft tissue infection. J Antimicrob Chemother.

[6] Stevens DL, Bisno AL, Chambers HF, Dellinger EP, Goldstein EJ, Gorbach SL, Hirschmann JV, Kaplan SL, Montoya JG, Wade JC (2014). Infectious Diseases Society of America. Practice guidelines for the diagnosis and management of skin and soft tissue infections: 2014 update by the Infectious Diseases Society of America. Clin Infect Dis.

[7] Coburn B, Morris AM, Tomlinson G, Detsky AS (2012). Does this adult patient with suspected bacteremia require blood cultures?. JAMA.

[8] Perl B, Gottehrer NP, Raveh D, Schlesinger Y, Rudensky B, Yinnon AM (1999). Cost-effectiveness of blood cultures for adult patients with cellulitis. Clin Infect Dis.

[9] Paolo WF, Poreda AR, Grant W, Scordino D, Wojcik S (2013). Blood culture results do not affect treatment in complicated cellulitis. J Emerg Med.

[10] Gunderson CG, Martinello RA (2012). A systematic review of bacteremias in cellulitis and erysipelas. J Inf Secur.

[11] Garau J, Ostermann H, Medina J, Avila M, McBride K, Blasi F, REACH study group (2013). Current management of patients hospitalized with complicated skin and soft tissue infections across Europe (2010-2011): assessment of clinical practice patterns and real-life effectiveness of antibiotics from the REACH study. Clin Microbiol Infect.

[12] van Daalen FV, Kallen MC, van den Bosch CMA, Hulscher MEJL, Geerlings SE, Prins JM (2017). Clinical condition and comorbidity as determinants for blood culture positivity in patients with skin and soft-tissue infections. Eur J Clin Microbiol Infect Dis.

[13] Lipsky BA, Kollef MH, Miller LG, Sun X, Johannes RS, Tabak YP (2010). Predicting bacteremia among patients hospitalized for skin and skin-structure infections: derivation and validation of a risk score. Infect Control Hosp Epidemiol.

[14] Jaaskelainen IH, Hagberg L, From J, Schyman T, Lehtola L, Jarvinen A (2016). Treatment of complicated skin and skin structure infections in areas with low incidence of antibiotic resistance-a retrospective population based study from Finland and Sweden. Clin Microbiol Infect.

[15] Jaaskelainen IH, Hagberg L, Forsblom E, Jarvinen A (2017). Factors associated with time to clinical stability in complicated skin and skin structure infections. Clin Microbiol Infect.

[16] Jaakola S, Lyytikäinen O, Rimhanen-Finne R, Salmenlinna S, Vuopio J, Roivainen M, Löflund J, Kuusi M, Ruutu P (2012) Tartuntataudit Suomessa 2011 [Infectious Diseases in Finland 2011]. Terveyden ja Hyvinvoinnin Laitos [National Institute for Health and Welfare]. Available at: http://urn.fi/URN:ISBN:978-952-245-658-8

[17] European Centre for Disease Prevention and Control (2012) Antimicrobial resistance surveillance in Europe 2011. Annual Report of the European Antimicrobial Resistance Surveillance Network (EARS-Net). ECDC, Stockholm

[18] Jaaskelainen IH, Hagberg L, Schyman T, Jarvinen A (2018). A potential benefit from infectious disease specialist and stationary ward in rational antibiotic therapy of complicated skin and skin structure infections. Infect Dis (Lond).

[19] Blackberg A, Trell K, Rasmussen M (2015). Erysipelas, a large retrospective study of aetiology and clinical presentation. BMC Infect Dis.

[20] Bauer S, Aubert CE, Richli M, Chuard C (2016). Blood cultures in the evaluation of uncomplicated cellulitis. Eur J Intern Med.

[21] Peralta G, Padron E, Roiz MP, De Benito I, Garrido JC, Talledo F, Rodriguez-Lera MJ, Ansorena L, Sanchez MB (2006). Risk factors for bacteremia in patients with limb cellulitis. Eur J Clin Microbiol Infect Dis.

[22] Eron LJ, Lipsky BA (2006). Use of cultures in cellulitis: when, how, and why?. Eur J Clin Microbiol Infect Dis.

